# Exercise, but Not Metformin Prevents Loss of Muscle Function Due to Doxorubicin in Mice Using an In Situ Method

**DOI:** 10.3390/ijms22179163

**Published:** 2021-08-25

**Authors:** Amy D. Mackay, Erik D. Marchant, Makensie Louw, David M. Thomson, Chad R. Hancock

**Affiliations:** 1 Nutrition, Dietetics, and Food Science, Brigham Young University, Provo, UT 84602, USA; amydmackay@gmail.com (A.D.M.); marchanterik@gmail.com (E.D.M.); kensiejoann@gmail.com (M.L.); 2 Cell Biology and Physiology, Brigham Young University, Provo, UT 84602, USA; david_Thomson@byu.edu

**Keywords:** skeletal muscle, chemotherapy, metformin, exercise, mitochondria, muscle contractions

## Abstract

Though effective in treating various types of cancer, the chemotherapeutic doxorubicin (DOX) is associated with skeletal muscle wasting and fatigue. The purpose of this study was to assess muscle function in situ following DOX administration in mice. Furthermore, pre-treatments with exercise (EX) or metformin (MET) were used in an attempt to preserve muscle function following DOX. Mice were assigned to the following groups: control, DOX, DOX + EX, or DOX + MET, and were given a single injection of DOX (15 mg/kg) or saline 3 days prior to sacrifice. Preceding the DOX injection, DOX + EX mice performed 60 min/day of running for 5 days, while DOX + MET mice received 5 daily oral doses of 500 mg/kg MET. Gastrocnemius–plantaris–soleus complex function was assessed in situ via direct stimulation of the sciatic nerve. DOX treatment increased time to half-relaxation following contractions, indicating impaired recovery (*p* < 0.05). Interestingly, EX prevented any increase in half-relaxation time, while MET did not. An impaired relaxation rate was associated with a reduction in SERCA1 protein content (*p* = 0.07) and AMPK phosphorylation (*p* < 0.05). There were no differences between groups in force production or mitochondrial respiration. These results suggest that EX, but not MET may be an effective strategy for the prevention of muscle fatigue following DOX administration in mice.

## 1. Introduction

The chemotherapeutic doxorubicin (DOX) is used clinically to treat a variety of solid tumor and leukemia cancers by intercalating DNA and inhibiting replication [1]. DOX use is known to cause cardiomyopathy, which can lead to acute left ventricular failure and congestive heart failure. In addition to the effects on cardiac muscle, DOX accumulates in skeletal and smooth muscle causing long-term side effects including severe fatigue and muscle loss [2,3,4]. Additional research into the mechanism of action of DOX on muscle function and potential strategies to mitigate toxicity is therefore warranted.

To date, most of the research on the effect of DOX on muscle has focused on issues related to cardiomyopathy [5,6,7,8,9,10,11,12]. However, maintaining skeletal muscle function is imperative for maintaining strength and mobility and improving functional ability during and after chemotherapy treatment. Strategies that mitigate the damaging side effects of DOX on muscle without interfering with its chemotherapeutic properties could improve overall patient quality of life.

Nearly 90% of patients receiving chemotherapy experience significant fatigue which impacts their ability to complete daily activities [2,13]. DOX is known to increase oxidative stress and activate proteolytic pathways within skeletal muscle in a rat model, suggesting that DOX accumulation in skeletal muscle contributes to overall fatigue and muscle weakness [14,15,16]. Additionally, ex vivo experiments show that DOX causes a decrease in muscle force production in rats [2,8,9,17,18,19,20]. In this study, we analyzed muscle function of the gastrocnemius–plantaris–soleus (GPS) complex in mice using an in situ approach. This method allows muscle function to be evaluated under intact blood flow conditions while muscle contractions are maximally activated via sciatic nerve stimulation [21]. Very little research has been done on evaluating muscle function following DOX treatment using an in situ approach.

Employing strategies such as exercise or the anti-diabetic drug metformin (MET) may limit skeletal muscle damage and promote improved quality of life for patients receiving DOX treatment. Patients with breast cancer that performed aerobic or resistance exercise while receiving adjuvant chemotherapy had reduced fatigue levels, improved aerobic capacity, improved muscle strength, and attenuated loss of bone mineral density compared to controls [22,23]. Recent studies in animal models show that exercise (EX) with DOX treatment limits skeletal muscle proteolysis [14,15,24], maintains mitochondrial respiration [25,26], and preserves muscle function [18]. The anti-diabetic drug metformin (MET) has also been investigated as a potential co-treatment to enhance the effectiveness of chemotherapy regimens [27]. Emerging literature in cell culture and animal models suggests that MET may decrease the toxic side effects of DOX in cardiac cells [28,29,30,31,32,33,34]. Furthermore, the effectiveness of MET in enhancing the chemotherapeutic effects of DOX and other chemotherapeutic agents has been the subject of recent clinical trials [35,36]. However, the effects of MET and DOX on skeletal muscle function have not yet been studied.

The purpose of this study is to determine if EX or MET treatment can limit skeletal muscle dysfunction due to DOX treatment. The negative effects of DOX treatment on skeletal muscle function were analyzed with or without EX or MET treatments in the GPS complex using an in situ approach. In addition, we investigated the potential protective mechanisms of action of EX or MET by analyzing mitochondrial respiratory capacity, hydrogen peroxide production, 5′ AMP-activated protein kinase (AMPK) phosphorylation, and sarco/endoplasmic reticulum Ca^2+^-ATPase 1 (SERCA1) protein levels.

## 2. Results

### 2.1. DOX Treatment Causes Severe Body Weight Loss at 3 Days

To determine the effects of DOX on muscle function and the potential benefit of exercise or metformin as co-treatments, a single bolus of DOX was administered to 6-week-old mice. Mice were euthanized 3 days post-DOX treatment. Consistent with previous studies, DOX treatment caused a significant reduction in body weight (Table 1). All groups had similar starting body weight (BW), and groups that received DOX lost a significant amount of weight (*p* < 0.0001). Exercise with DOX tended to limit the loss of body weight compared to DOX alone (*p* = 0.08). DOX treatment also had a significant effect on GPS muscle weight (Control 135.6 ± 4.7 mg vs. all DOX groups 116.4 ± 2.5 mg; *p* < 0.001). When calf weight was expressed as a percent of BW, there wasn’t a significant benefit of EX or MET with DOX compared to DOX alone. If anything, there may have been additional muscle loss relative to body weight in the DOX + MET compared to DOX alone (Table 1).

### 2.2. DOX Treatment and Muscle Function of the GPS Complex

**Figure 1:**
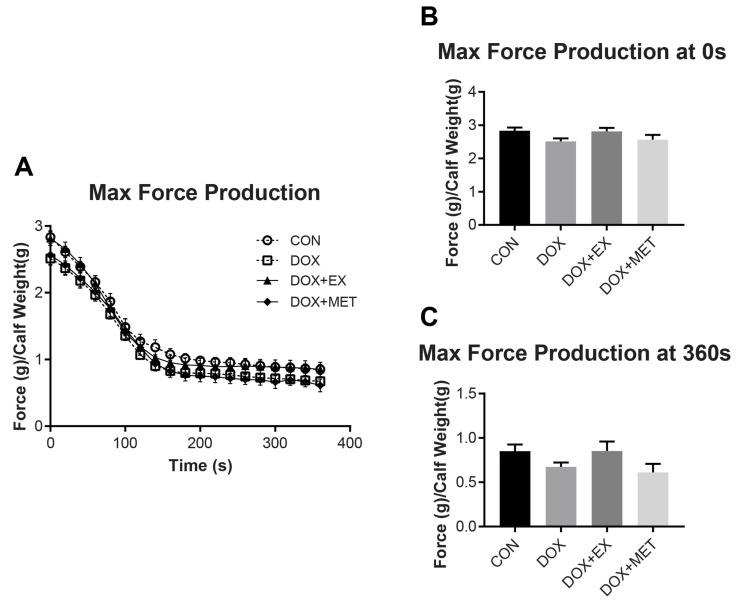
The force Production of GPS complex over 6 min fatiguing protocol. (**A**) the force over 6 min protocol. (**B**) The force at time point 0 s. (**C**) The force at time point 360 s. Data are represented as mean ± SEM (*n* = 7–10). No statistical differences were reported.

Maximal force production of the GPS complex over a 6 min fatigue protocol is shown in Figure 1A. DOX treatment did not cause a change in force production at any time point during the fatiguing protocol. Likewise, we did not observe a benefit on maximal force production with either EX or MET treatments in combination with DOX. Representations of force produced are shown at time 0 s (Figure 1B) and time 360 s (Figure 1C). The integrated force was also measured as the sum of the force produced in each contraction over the 6 min fatiguing protocol, and no differences were found (not shown).

**Figure 2:**
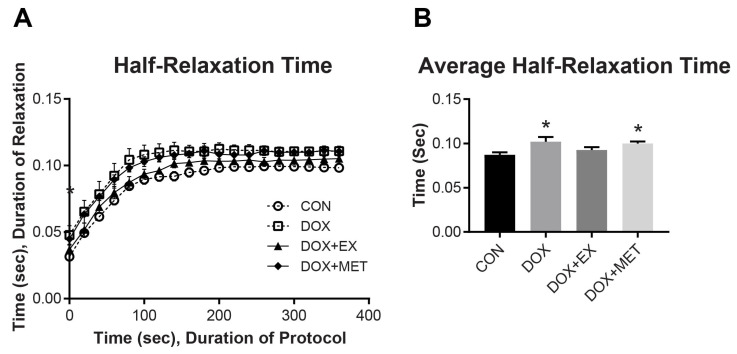
The half-relaxation time of GPS complex over 6 min fatiguing protocol. (**A**) Half-relaxation time over 6 min protocol. (**B**) The average half-relaxation time over the entire 6 min fatiguing protocol. Data are represented as mean ± SEM (*n* = 7–10). Statistical differences (*p* < 0.05) from CON are indicated by an asterisk (*).

In addition to fatigue and max force production, we evaluated muscle relaxation times to better understand the effect of DOX on muscle energetics and recovery. DOX increased the time for half-relaxation, indicating slower muscle recovery (Figure 2). DOX slowed half-relaxation time by an average of 17% compared to CON (*p* < 0.05 averaged over the whole contraction period). The combination of EX with DOX treatment resulted in relaxation times that were not different from CON (Figure 2B). The combination of MET with DOX did not result in an improvement in relaxation times compared to DOX and was 15% slower than control (*p* < 0.05). These results indicate that EX, but not MET may have provided some protection against DOX-induced slowing of half-relaxation time. 

### 2.3. DOX Treatment Does Not Impair Mitochondrial Respiration

**Figure 3:**
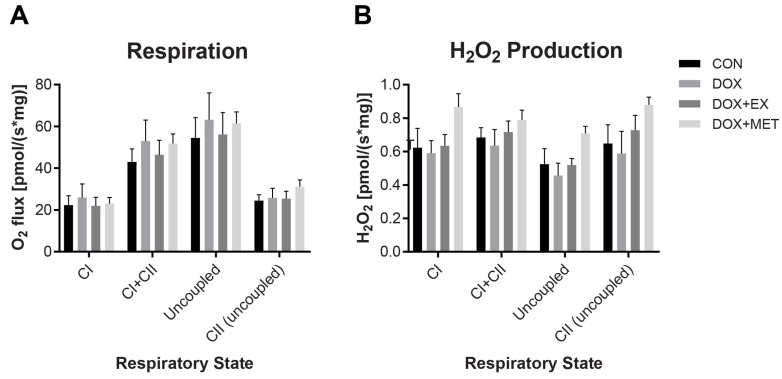
The respiratory capacity and H_2_O_2_ production using permeabilized fibers from red gastrocnemius muscle. (**A**) The respiratory capacity of fibers following the addition of glutamate, malate, and ADP (CI), succinate (CI + CII), FCCP (Uncoupled) and rotenone (CII (uncoupled)) (*n* = 6–9). (**B**) H_2_O_2_ production under the same conditions as respiratory capacity measurements (*n* = 3–6). No statistical differences are reported.

High-resolution respirometry was used to investigate if the DOX-induced impairment in half-relaxation time was associated with a change in mitochondrial respiratory capacity and hydrogen peroxide production. There was no difference in respiration rates or hydrogen peroxide production among any groups (Figure 3). Complex I supported respiration was measured after the addition of glutamate, malate, and ADP. Complex I + II measurements were taken after the addition of succinate. FCCP was added to uncouple respiration, and uncoupled complex II was measured after adding the complex I inhibitor rotenone. No differences in either respiration or hydrogen peroxide measurements suggest that mitochondrial function is not disrupted at this time point in response to DOX treatment.

### 2.4. DOX Treatment Causes a Reduction in AMPK Phosphorylation

**Figure 4:**
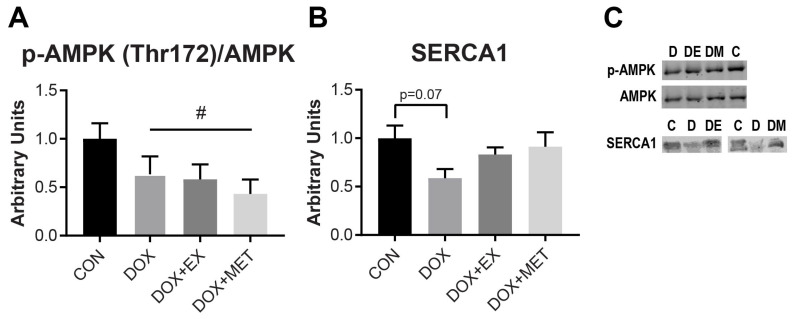
AMPK phosphorylation and SERCA1 protein expression measured in gastrocnemius muscle via Western blot. (**A**) AMPK phosphorylation (Thr172) (*n* = 5). (**B**) SERCA1 protein expression (*n* = 5). (**C**) Representative Western blots. Main effect of DOX (*p* < 0.05) is indicated by #.

5′ AMP-activated protein kinase (AMPK) is a cellular energy sensor that responds to changes in energy state and is active in its phosphorylated state [37]. Therefore, we sought to determine if we could observe differences in AMPK activation by measuring AMPK phosphorylation. Though there were no changes in mitochondrial respiration following DOX treatment, we observed a decrease in AMPK phosphorylation following DOX. All animals treated with DOX had lower AMPK phosphorylation (~40%) compared to control animals (*p* < 0.05). Neither EX nor MET interventions were able to prevent the effect of DOX (Figure 4A). Thus, the observed improvement in relaxation following exercise does not appear to be associated with an improvement in the energy state of the muscle, as reflected by AMPK phosphorylation.

### 2.5. DOX Treatment Causes a Reduction in SERCA1 Protein Expression

The sarco/endoplasmic reticulum Ca^2+^-ATPase (SERCA) is responsible for the transport of Ca^2+^ into the sarcoplasmic reticulum following muscle fiber contractions. Because SERCA is responsible for the clearance of Ca^2+^ from the cytosol and is necessary for muscle relaxation, we sought to evaluate SERCA protein expression in the gastrocnemius muscle. In mice, the calf muscle complex is predominantly composed of fast-twitch muscle fibers (~93% fast-twitch and 7% slow-twitch [38]), which express the SERCA1 isoform [39]. Following DOX treatment, SERCA1 protein expression tended to be reduced in the gastrocnemius muscle (*p* = 0.07). SERCA1 expression was not different from control with the combination of EX or MET with DOX (Figure 4B). The apparent changes in SERCA1 expression with DOX are consistent with the observed slowing of muscle relaxation. In addition, EX or MET seems to have prevented a decline in SERCA1 expression, though only the combination of EX with DOX was associated with an improvement in relaxation rates compared to DOX treatment alone.

## 3. Discussion

The purpose of this study was to characterize skeletal muscle dysfunction following DOX treatment and to determine if EX or MET treatment can prevent DOX-induced impairments in skeletal muscle function. The one indication of muscle dysfunction we observed with DOX treatment was an increase in GPS complex half-relaxation time. The slowing of relaxation time was associated with a decrease in SERCA1 protein expression. Interestingly, there was no effect of DOX on maximal muscle twitch force, or on mitochondrial respiration and hydrogen peroxide production. EX pre-treatment prevented a slowing of half-relaxation time following DOX treatment, but MET treatment had no effect. These results suggest that EX, but not MET, may provide some protection against the damaging effects of DOX treatment.

To our knowledge, this study is the first to determine the effects of DOX treatment on muscle function using in situ force measurements. The benefit of using an in situ method to measure force production is that the muscle is left intact, allowing for continual blood flow during muscle contractions and full contractions are elicited via direct stimulation of the sciatic nerve. The measurements of force production made in our study indicate that DOX treatment did not cause a decrease in specific force production, which is different from previous reports [9,17,20]. One possible explanation for this discrepancy is that muscle dysfunction has been shown to progress continually until at least 5 days post-DOX treatment [8]. Therefore, at the 3-day time point used in this study, it is possible that skeletal muscle dysfunction may not have fully developed. Despite the lack of a reduction in muscle force production, the dose and length of DOX treatment used are still relevant to DOX-induced muscle atrophy as indicated by a reduction in muscle size and overall body weight.

A second possible explanation is that DOX has been shown to have a greater effect on slow-twitch, highly oxidative type I muscle fibers than on fast-twitch, glycolytic type II muscle fibers. In this study, we analyzed the GPS complex in the mouse, which is composed of about 93% fast-twitch fibers [38]. Most of the other muscle function studies with DOX treatment have been carried out in a rat model [8,9,17,18,19] using the soleus (SOL) and extensor digitorum longus (EDL). The SOL in rats is composed of ~80% slow-twitch fibers, while the EDL is composed of ~5% slow-twitch fibers [40]. In these studies, DOX treatment has been shown to have a more profound effect on the slow-twitch SOL muscle than the fast-twitch EDL muscle [8,9,17,18]. The only functional impairment due to DOX treatment we observed was a slowing of GPS muscle relaxation. This is consistent with the milder effects of DOX on muscle composed of predominantly fast-twitch muscle fibers.

A third potential explanation for why we did not observe a reduction in muscle force following DOX treatment, in contrast to other studies, could be that there are differences in oxygen delivery between in situ and ex vivo muscle preparations. Previous reports suggest that differences in maximal isometric force may be observed between in situ and ex vivo preparations, and that these differences can be attributed to the size of muscle strips used in ex vivo methods, with larger strips exhibiting greater fatigue due to increased oxygen diffusion distance and hypoxia [41]. Therefore, it is possible that DOX-induced changes in muscle force production over a fatiguing protocol could be influenced by the method of muscle preparation and that an in situ method may be preferable because blood flow is still intact.

The data from our study show that DOX slowed half-relaxation time, an effect that was prevented in the DOX + EX group but not the DOX + MET group. Half-relaxation time is the time required for the muscle to relax by 50% following the completion of contraction. As the muscle fatigues, the time to half-relaxation increases. Because the muscle was given time to recover between contractions, the impaired relaxation rate did not affect the peak force production of the muscle. Previous work done by Hancock et al. and others [21,42,43] suggests that the half-relaxation time is closely associated with changes to the energy state of the muscle. This is due to the energetically expensive process of calcium sequestration from the cytosol to the sarcoplasmic reticulum. To better understand the mechanism behind how half-relaxation time was impaired, we evaluated mitochondrial respiration and SERCA protein expression.

In the present study, DOX did not affect mitochondrial respiratory capacity, which is different from previous reports [25,26,44]. Specifically, Gilliam et al. reported an acute effect of DOX on mitochondrial function (2 h post-treatment), followed by a recovery period (24 h post-treatment) and an eventual reduction of respiratory function (3 days post-treatment) [25]. In the present study, there was no change in mitochondrial respiration at the 3 day time point, which may suggest that mice have a different period of recovery from DOX treatment than rats do. We anticipate that we might observe the impact of DOX on respiratory function with a more acute treatment or a longer one in a mouse model. Interestingly, Tarpey et al. reported decrements in mitochondrial respiration in mice at the 3-day time point, which is different from our results. It should be noted, however, that they used a higher dose of DOX (20 mg/kg) than that used in our study (15 mg/kg) [44]. Our data suggest that mitochondrial dysfunction may not be required to induce impairment in muscle function, as shown with slowed half-relaxation time. However, it should be noted that we did not evaluate respiratory kinetics, such as ADP sensitivity and electron conductance through the electron transport system, which has been previously reported to be affected by DOX treatment [44]. It is possible that respiratory kinetics were altered and that these changes could have affected skeletal muscle energy status and half-relaxation time.

Though there was no apparent change in mitochondrial respiration, suggesting that the bioenergetic status of the muscle was intact, we observed a reduction in AMPK phosphorylation following DOX treatment, as well as a reduction in SERCA1 protein expression. It is important to consider that the gastrocnemius muscle used for the measurement of AMPK phosphorylation was excised immediately following the metabolic stress induced by a fatiguing stimulation protocol. Thus, the reduction in AMPK phosphorylation may suggest that DOX impairs the ability of the muscle to respond to energetic stress. These results are consistent with a previous study in rats where DOX was given in the same dose (15 mg/kg) and for the same length of time (3 days), which resulted in a reduction in AMPK phosphorylation in skeletal muscle [45]. Furthermore, it has been shown in cardiac tissue that DOX can induce metabolic stress and a rise in AMP:ATP ratios, while still inhibiting AMPK phosphorylation [46], though there are admittedly mixed results that may depend on the dose and length of treatment [47]. Regardless, several studies suggest that AMPK activation is impaired by DOX, and that AMPK activation may be a potential therapeutic target for protecting cardiac muscle, as discussed in a recent review by Timm and Tyler [47]. To our knowledge, the mechanism for how AMPK activation may be suppressed following DOX treatment has not been characterized. The reduction in phosphorylated AMPK was not rescued by exercise or metformin treatment. 

Another interesting finding in our study was that SERCA1 protein expression was decreased 3 days after DOX administration but was no different from control when combined with EX or MET, indicating that SERCA1 expression may be protected by EX or MET. Due to the role that SERCA1 plays in regulating relaxation rates in fast-twitch skeletal muscle, it is possible that this decrease in protein was responsible for the increase in half-relaxation time observed in situ. Previous research has shown that SERCA2 protein expression, the isoform found in slow-twitch and heart muscle, is decreased in rat and rabbit cardiac muscle following DOX administration [10,48]. Our results suggest that future research should investigate the effects of DOX on SERCA protein expression, as well as other regulators of Ca^2+^ homeostasis.

Our study presents the finding that DOX induces a slowing of muscle relaxation time in the GPS complex of mice, which is prevented by EX but not MET. Interestingly, this slowing of relaxation time was not associated with a change in muscle force production or mitochondrial function but was associated with a reduction in SERCA1 protein expression and AMPK phosphorylation. This study is valuable because it adds to the understanding of how DOX treatment can impact skeletal muscle function. In addition, exercise may improve functional outcomes following DOX treatment by preventing disruption in calcium homeostasis.

## 4. Methods

### 4.1. Animal Care

All animal care and experimental procedures were approved by the Institutional Animal Care and Use Committee of Brigham Young University on 10 July 2012 (Protocol #12-0703). All experiments were carried out in compliance with the Animal Welfare Act. The animals used in this study were also part of a separate analysis of iron dysregulation caused by DOX treatment [49]. Five-week-old C57BL/6 mice were fed standard chow (8064 Harlan Teklad/Envigo, Indianapolis, IN, USA) and water ad libitum and housed in a temperature-controlled environment (21–22 °C) with a 12 h light/dark cycle.

Mice were randomly assigned to 4 groups: control (CON), doxorubicin (DOX), doxorubicin + exercise (DOX + EX), or doxorubicin + metformin (DOX + MET). Mice with DOX treatments received a single 15 mg/kg intraperitoneal injection 3 days prior to sacrifice. This dose has previously been shown to be sufficient to cause muscle dysfunction (ex vivo analysis) in a rodent model [8,18,19]. Though previous studies have used doses as high as 20 mg/kg, this dose has been shown to cause death in 60–70% of wild-type mice within just one week [50,51,52]. Thus, we chose to use a dose that was potent enough to induce muscle wasting and weight loss (16% loss on average) but would not lead to the death of animals. The mice were sacrificed 3 days after DOX injection. Mice in the exercise treatment groups were acclimated to the treadmill on days 1–3 (5–10 m/min for 10 min/day), performed a maximum running test on day 4, and allowed to rest on days 5–6. On days 7–11, mice performed 60 min of running at ~70% max speed. DOX (44583 Sigma-Aldrich, St. Louis, MO, USA) treatments were given on the final day of exercise, after completion of the exercise bout. Mice with MET (PHR1084 Sigma-Aldrich, St. Louis, MO, USA) treatments were given 5 daily doses at 500 mg/kg body weight via oral gavage beginning two days before DOX treatment. Control mice received saline treatments to mimic MET and DOX treatments. 

### 4.2. In Situ Muscle Function

In situ measurements of contractile function and fatigue of the gastrocnemius—plantaris—soleus (GPS) complex were made as described previously [21,53]. Briefly, mice were anesthetized with 2.5–3.5% vaporized isoflurane with supplemental oxygen. The calf muscle complex was isolated by tying off the Achilles tendon with a small portion of the calcaneus bone. The Achilles tendon was then attached to the lever arm of a dual-mode lever system (305C Aurora Scientific, Aurora, ON, Canada) and held in place during the contraction bout. Tetanic contractions of the GPS were elicited by direct stimulation of the sciatic nerve (2–4 V) for 6 min using a Grass S88X stimulator (0.5 Hz train frequency, 100 ms train duration, 150 Hz pulse frequency). Results were analyzed using ASI 610A Dynamic Muscle Control V5.500 software. Data were analyzed for peak force, and half-relaxation times.

### 4.3. Mitochondrial Respiration

Mitochondrial respiration was assessed as previously described [54]. Briefly, small portions of gastrocnemius muscle (~6 mg) were blotted, weighed, and kept in ice-cold BIOPS buffer (60 mM K-MES, 35 mM KCl, 7.23 mM K_2_EGTA, 2.77 mM CaK_2_EGTA, 20 mM imidazole, 0.5 mM DTT, 20 mM taurine, 5.7 mM ATP, 15 mM PCr, and 6.56 mM MgCl_2_). Muscle fibers were teased apart using forceps to partially separate fibers without completely separating them from the fiber bundle. Fibers were then rotated for 30 min at 4 °C in BIOPS buffer supplemented with 20 μg/mL saponin (47036 Sigma-Aldrich, St. Louis, MO, USA) to permeabilize the cell membranes. Following permeabilization, muscle fibers were rinsed for 15 min with ice-cold MiR05 buffer (110 mM sucrose, 60 mM potassium lactobionate, 2 mM MgCl_2_, 20 mM taurine, 10 mM KH_2_PO_4_, 0.5 mM EGTA, 20 mM HEPES, and 1 g/L bovine serum albumin). Following this, fibers were placed inside the chambers of the Oxygraph-2k (OROBOROS INSTRUMENTS, Innsbruck, Austria) to measure respiration. MiR05 buffer was used in the O2k, and the chamber temperature was set at 25 °C, with stir bars spinning at 750 revolutions/min. Oxygen was added to the chambers prior to sealing them off, ensuring that the concentration of oxygen in the buffer stayed between ~450 and 200 pmol O_2_. Substrates were added to the chambers with the following titration protocol: First, glutamate (2 mM; G1626 Sigma-Aldrich, St. Louis, MO, USA), malate (10 mM; M1000 Sigma-Aldrich, St. Louis, MO, USA) and ADP (2.5 mM; 117,105 EMD-Millipore, Burlington, MA, USA) were added to stimulate Complex I-supported respiration (CI). Second, cytochrome C (10 µM; C3131 Sigma-Aldrich, St. Louis, MO, USA) was added to ensure mitochondrial membrane integrity, and any data with an increase in respiration more than 10% were discarded. Third, succinate (10 mM; S2378 Sigma-Aldrich, St. Louis, MO, USA) was added to stimulate maximal coupled respiration (CI + CII). Maximum uncoupled respiration was measured using FCCP (0.5 µM steps; C2920 Sigma-Aldrich, St. Louis, MO, USA). Complex I was then inhibited with rotenone (0.5 µM; R8875 Sigma-Aldrich, St. Louis, MO, USA). Finally, all respiration was inhibited with antimycin A (2.5 µM; A8674 Sigma-Aldrich, St. Louis, MO, USA) and resulting residual oxygen consumption was subtracted from the data set.

### 4.4. Hydrogen Peroxide Production

Rates of H_2_O_2_ emission were assessed using the O2k fluorimetry module. Prior to introducing tissue into the chamber and titrating the previously mentioned complex-stimulating substrates, Amplex Red (10 µM; A12222 Invitrogen, Waltham, MA, USA) and horseradish peroxidase (1 U/mL; P8250 Sigma-Aldrich, St. Louis, MO, USA) were added to each chamber. H_2_O_2_ (stabilized by 10 µM HCl) was then titrated into the chamber in 0.1 µM steps (final concentration) to create a standard curve. During the oxygen consumption analysis, H_2_O_2_ production was measured. Amplex Red reacted with H_2_O_2_ to form resorufin using horseradish peroxidase as the catalyst. Resorufin concentration was measured via fluorescence with 563 nm excitation and 587 nm emission wavelengths.

### 4.5. Western Blotting 

Gastrocnemius muscle was homogenized at a ratio of 19 μL homogenization buffer (50 mM Tris-HCl, 250 mM mannitol, 1% triton x-100, 50 mM β-glycerophosphate, 1 mM EDTA, 1 mM EGTA, 1 mM dithiothreitol, 50 mM NaF, 5 mM sodium pyrophosphate, 1 mM sodium orthovanadate, 5 μg/mL soybean trypsin inhibitor, 1 μL/mL aprotinin, 1 μL/mL pepstatin; pH 7.4) to 1 mg tissue. Cell membranes were disrupted with 3 freeze-thaw cycles, and total protein content was quantified using a bicinchoninic acid protein assay (786-571 G-Biosciences, St. Louis, MO, USA). Samples were made with equal protein concentrations in Laemmli buffer and resolved on SDS-polyacrylamide gels. Following electrophoresis, the proteins were transferred to nitrocellulose membranes (#1620112 BIO-RAD, Hercules, CA, USA) in ice-cold transfer buffer. Each membrane was then stained for total protein using Ponceau S BioReagent (P3504 Sigma-Aldrich, St. Louis, MO, USA) and quantified using Image-J software to ensure equal protein loading. The membranes were blocked for 60 min in either 5% non-fat dry milk or bovine serum albumin (BSA) in tris-buffered saline, 0.1% TWEEN 20 (TBST). Following blocking, the membranes were incubated overnight with primary antibodies at 4 °C. The following primary antibodies were diluted in TBST with 1% BSA: AMPKα (1:4000; #2532 Cell-Signaling Technology Inc., Danvers, MA, USA), Phospho-AMPKα (Thr 172) (1:4000; #2535 Cell-Signaling Technology Inc., Danvers, MA, USA), and ATP2A1/SERCA1 (1:500; #12293 Cell-Signaling Technology, Inc., Danvers, MA, USA). Membranes were washed in TBST 4 times for 5 min, and then incubated for 1 h at ambient temperature in the following secondary antibodies diluted in TBST with 1% BSA: Anti-mouse IgG secondary antibody (1:10,000; 629–65,010; IRDye, Lincoln, NE, USA) and Anti-rabbit IgG secondary antibody (1:10,000; 629–32,213; IRDye, Lincoln, NE, USA). Membranes were washed again in TBST 4 times for 5 min and imaged using direct infrared imaging (Odyssey CLx, LICOR Biosciences, Lincoln, NE, USA). Samples from each group were averaged and normalized to control samples.

### 4.6. Statistics

Differences between treatment groups were determined via one-way ANOVA and a Tukey HSD post hoc analysis. Additionally, a main effect of DOX treatment is reported for change in body weight and AMPK phosphorylation, which was determined by a student’s *t*-test, with all mice receiving DOX compared to control. All significant differences were tested with an alpha level of 0.05. Animals that were assigned to receive DOX treatment but did not lose more than about 3% of body weight during the study were excluded because this was evidence that they did receive a proper DOX injection (the average body weight loss following DOX was about 14% and there were four animals excluded because they had an average of 4.1% increase in body weight). Furthermore, if animals from the control group lost more than about 3% of body weight over the course of the study, they were excluded from analysis because this indicated a systemic stress that was not consistent with the assigned group.

## Figures and Tables

**Table 1:** Average mouse body weight (BW) and calf weight (CW).

	CON (*n* = 23)	DOX (*n* = 20)	DOX + EX (*n* = 25)	DOX + MET (*n* = 19)
Starting BW (g)	19.8 ± 0.3	20.0 ± 0.3	20.3 ± 0.3	19.7 ± 0.4
Final BW (g)	20.2 ± 0.4 ^a^	16.7 ± 0.3 ^b^*	17.7 ± 0.4 ^b^*	17.0 ± 0.3 ^b^*
% Change in BW	2.2 ± 0.8 ^a^	−16.4 ± 0.9 ^b^*	−12.6 ± 1.4 ^b^*	−13.6 ± 1.1 ^b^*
Final CW (mg)	135.6 ± 4.7 ^a^	123.6 ± 5.7 ^ab^	119.5 ± 2.6 ^b^	108.5 ± 3.2 ^b^
CW as % of BW	0.65 ± 0.02 ^ab^	0.71 ± 0.02 ^a^	0.71 ± 0.02 ^a^	0.62 ± 0.02 ^b^

Average weights ± SEM. Values with different letters are significantly different (*p* < 0.05). An asterisk (*) indicates a difference from control with a *p*-value of <0.0001.
